# The genome sequence of the European pine marten, *Martes martes* (Linnaeus, 1758)

**DOI:** 10.12688/wellcomeopenres.22458.1

**Published:** 2024-06-21

**Authors:** David O'Brien, Inez Januszczak, Natural History Museum Genome Acquisition Lab, Darwin Tree of Life Barcoding collective, Wellcome Sanger Institute Tree of Life Management, Samples and Laboratory team, Wellcome Sanger Institute Scientific Operations: Sequencing Operations, Wellcome Sanger Institute Tree of Life Core Informatics team, Tree of Life Core Informatics collective, Darwin Tree of Life Consortium

**Affiliations:** 1 NatureScot, Inverness, Scotland, UK; 2 Natural History Museum, London, England, UK

**Keywords:** Martes martes, European pine marten, genome sequence, chromosomal, Carnivora

## Abstract

We present a genome assembly from an individual male *Martes martes* (the European pine marten; Chordata; Mammalia; Carnivora; Mustelidae). The genome sequence is 2,484.6 megabases in span. Most of the assembly is scaffolded into 20 chromosomal pseudomolecules, including the X and Y sex chromosomes. The mitochondrial genome has also been assembled and is 16.57 kilobases in length.

## Species taxonomy

Eukaryota; Opisthokonta; Metazoa; Eumetazoa; Bilateria; Deuterostomia; Chordata; Craniata; Vertebrata; Gnathostomata; Teleostomi; Euteleostomi; Sarcopterygii; Dipnotetrapodomorpha; Tetrapoda; Amniota; Mammalia; Theria; Eutheria; Boreoeutheria; Laurasiatheria; Carnivora; Caniformia; Musteloidea; Mustelidae; Guloninae; *Martes*; *Martes martes* (Linnaeus, 1758) (NCBI:txid29065).

## Background

**Figure 1.**
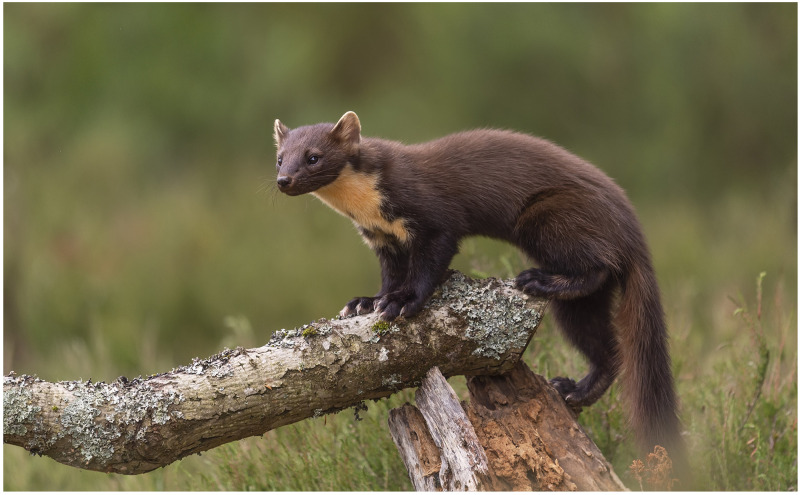
Photograph of *Martes martes* (not the specimen used for genome sequencing). Photograph by Caroline Legg.

The European pine marten, *Martes martes*, is one of Britain’s most iconic animals ( Figure 1). With distinct brown fur and a yellowish bib over the throat and chest, pine martens are one of the eight species of terrestrial mammalian carnivore native to the UK ( Sainsbury *et al.*, 2019). They are a small, nocturnal, semi-arboreal mustelid, typically inhabiting well-wooded areas; although they are known to use heathland and other open habitats in degraded landscapes ( Caryl *et al.*, 2012). Their body length ranges from 36 to 55 cm, with a tail length of 20–25 cm. Males are slightly larger than females, and their lifespan is listed as up to 18 years in the wild ( Harris & Yalden, 2008). Larger mammalian predators, such as red foxes, have been recorded to prey on pine martens, although not always as predominantly as assumed ( Sidorovich *et al.*, 2006). They mark their territory and home range with scat (faeces); which are used for monitoring purposes ( Trees for Life, no date).

Once prevalent throughout Britain, by the late 19th century the population was reduced to the north and west of the Scottish Highlands and small, isolated areas of northern England and north Wales ( Langley & Yalden, 1977). Reasons include deforestation, being hunted for fur as well as intensive predator control by gamekeepers ( Sainsbury *et al.*, 2019). The British population is estimated to have declined from around 145,000 in the Mesolithic to near extinction by 1915, with current numbers around 4,000 ( Maroo & Yalden, 2000; Mathews & Harrower, 2020). It is widely distributed across the western Palearctic ( Harris & Yalden, 2008).

Since the early 1980s, reintroductions have taken place (starting with Galloway Forest, southwest Scotland) ( Shaw & Livingstone, 1992), resulting in further pine marten expansion across the south of the Scotland. Reintroduction work has since continued; an example being in 2015 and 2016, 39 pine martens were translocated from Scotland to Wales, contributing to a now viable population ( McNicol *et al.*, 2020). The UK has one of the lowest forest covers in Europe; the latest National Statistics on woodland produced by Forest Research, released in June 2023, state that the 13% of the total land in the UK can be classified by woodland, with 19% of woodland found in Scotland ( Forest Research, 2023). The growth in population has led to pine marten being classed as Least Concern at a Scotland and GB, though despite increasing sightings, they are still listed as Critically Endangered in England and Wales ( Mathews & Harrower, 2020). Populations in northern England and north Wales are fragmented and small ( The Wildlife Trusts, no date) Continued legal protection combined with effective habitat restoration, reintroductions and headstarting will be key in maintaining the British pine marten population.

We present a chromosomally complete genome sequence for a male European pine marten ( *Martes martes*) as part of the Darwin Tree of Life Project. This project is a collaborative effort to sequence all named eukaryotic species in the Atlantic Archipelago, encompassing Britain and Ireland.

## Genome sequence report

The genome was sequenced from adult male *Martes martes* collected from Glen Carron, Scotland, UK (57.51, –5.21). A total of 35-fold coverage in Pacific Biosciences single-molecule HiFi long reads was generated. Primary assembly contigs were scaffolded with chromosome conformation Hi-C data. Manual assembly curation corrected 78 missing joins or mis-joins, reducing the scaffold number by 10.00%.

**Figure 2.**
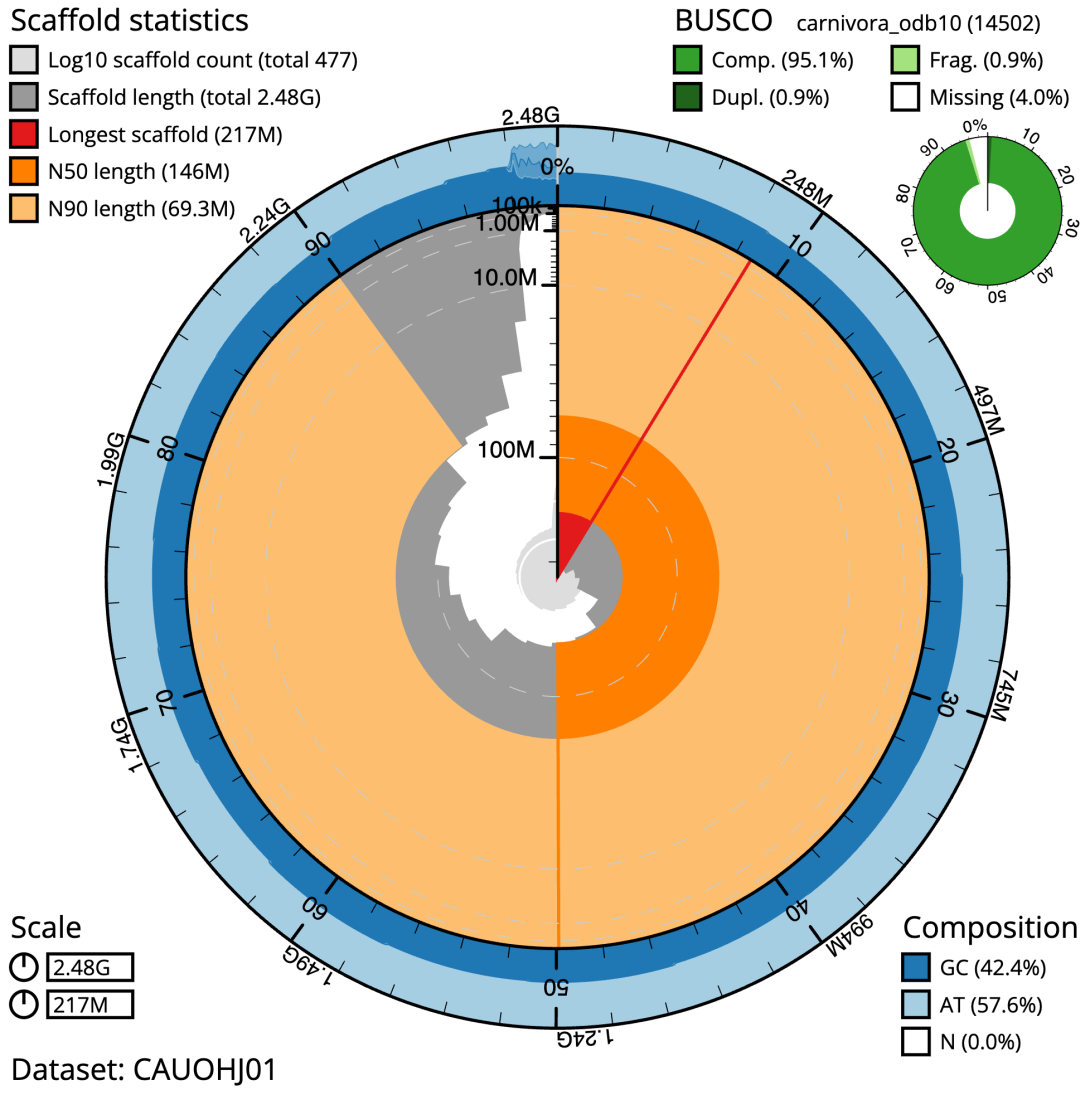
Genome assembly of *Martes martes*, mMarMar1.1: metrics. The BlobToolKit snail plot shows N50 metrics and BUSCO gene completeness. The main plot is divided into 1,000 size-ordered bins around the circumference with each bin representing 0.1% of the 2,484,650,161 bp assembly. The distribution of sequence lengths is shown in dark grey with the plot radius scaled to the longest sequence present in the assembly (217,416,870 bp, shown in red). Orange and pale-orange arcs show the N50 and N90 sequence lengths (146,289,188 and 69,260,160 bp), respectively. The pale grey spiral shows the cumulative sequence count on a log scale with white scale lines showing successive orders of magnitude. The blue and pale-blue area around the outside of the plot shows the distribution of GC, AT and N percentages in the same bins as the inner plot. A summary of complete, fragmented, duplicated and missing BUSCO genes in the carnivora_odb10 set is shown in the top right. An interactive version of this figure is available at https://blobtoolkit.genomehubs.org/view/Martes%20martes/dataset/CAUOHJ01/snail.

**Figure 3.**
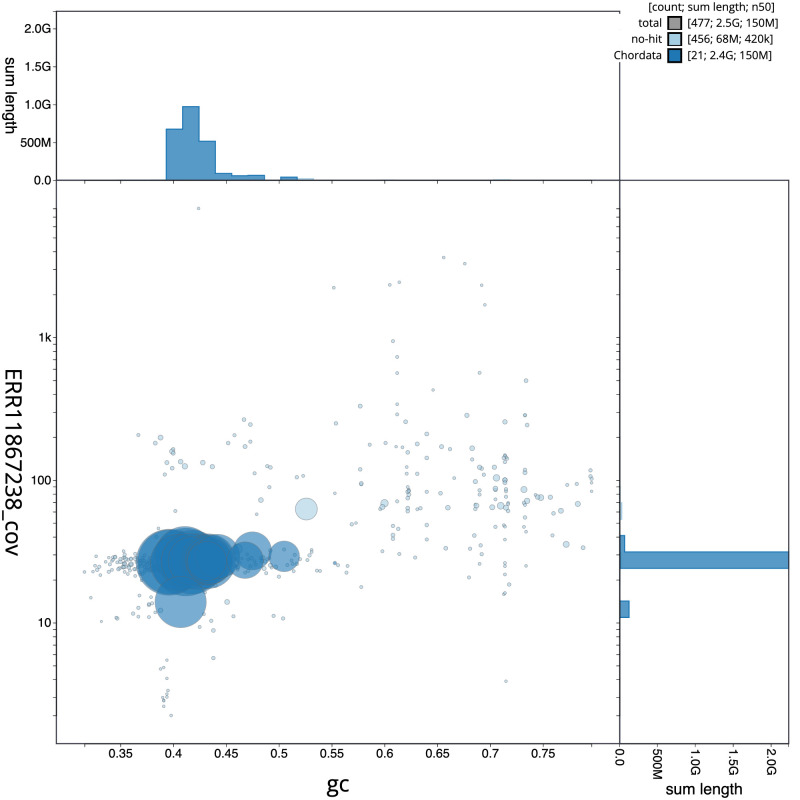
Genome assembly of *Martes martes*, mMarMar1.1: BlobToolKit GC-coverage plot. Sequences are coloured by phylum. Circles are sized in proportion to sequence length. Histograms show the distribution of sequence length sum along each axis. An interactive version of this figure is available at https://blobtoolkit.genomehubs.org/view/Martes%20martes/dataset/CAUOHJ01/blob.

**Figure 4.**
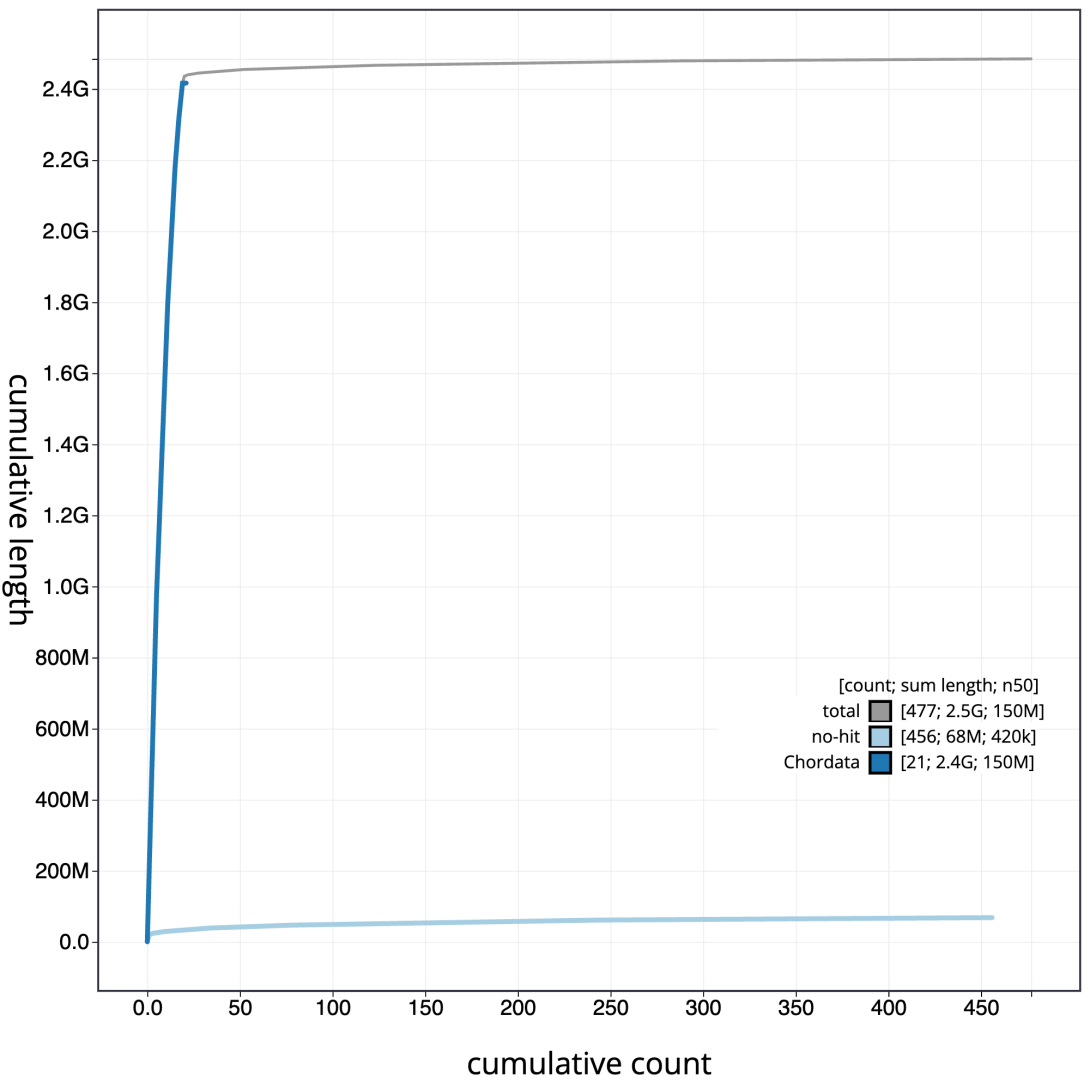
Genome assembly of *Martes martes* mMarMar1.1: BlobToolKit cumulative sequence plot. The grey line shows cumulative length for all sequences. Coloured lines show cumulative lengths of sequences assigned to each phylum using the buscogenes taxrule. An interactive version of this figure is available at https://blobtoolkit.genomehubs.org/view/Martes%20martes/dataset/CAUOHJ01/cumulative.

**Figure 5.**
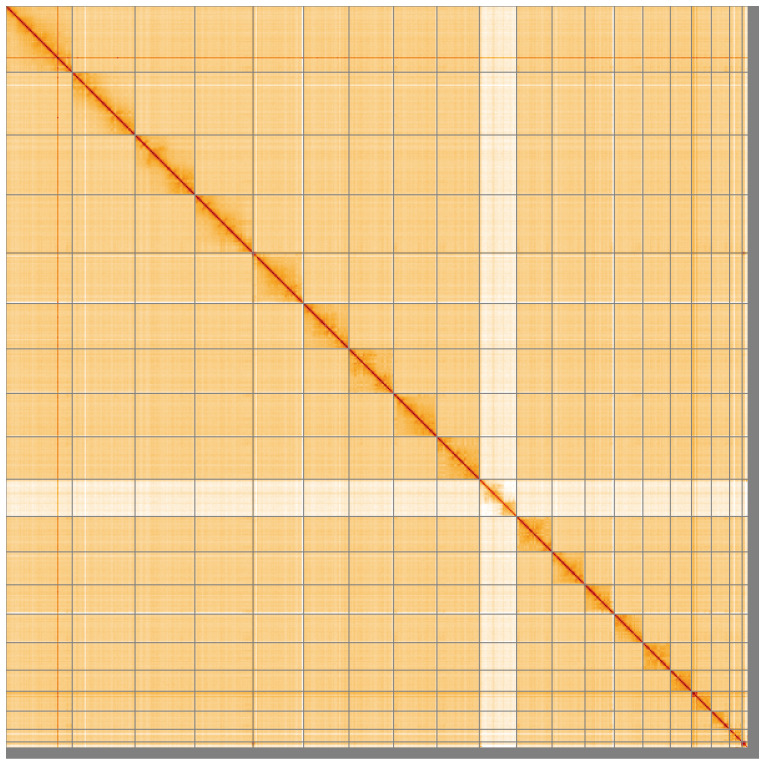
Genome assembly of *Martes martes* mMarMar1.1: Hi-C contact map of the mMarMar1.1 assembly, visualised using HiGlass. Chromosomes are shown in order of size from left to right and top to bottom. An interactive version of this figure may be viewed at https://genome-note-higlass.tol.sanger.ac.uk/l/?d=OH10xmpjSzCT8bLQ3J_c4w.

The final assembly has a total length of 2,484.6 Mb in 476 sequence scaffolds with a scaffold N50 of 146.3 Mb ( Table 1). The snail plot in Figure 2 provides a summary of the assembly statistics, while the distribution of assembly scaffolds on GC proportion and coverage is shown in Figure 3. The cumulative assembly plot in Figure 4 shows curves for subsets of scaffolds assigned to different phyla. Most (98.23%) of the assembly sequence was assigned to 20 chromosomal-level scaffolds, representing 18 autosomes and the X and Y sex chromosomes. Chromosome-scale scaffolds confirmed by the Hi-C data are named in order of size ( Figure 5; Table 2). Chromosomes X and Y were assigned by read coverage statistics and synteny to *Martes flavigula* (GCA_029410595.1). While not fully phased, the assembly deposited is of one haplotype. Contigs corresponding to the second haplotype have also been deposited. The mitochondrial genome was also assembled and can be found as a contig within the multifasta file of the genome submission.

**Table 1.** Genome data for *Martes martes*, mMarMar1.1.

Project accession data		
Assembly identifier	mMarMar1.1	
Species	*Martes martes*	
Specimen	mMarMar1	
NCBI taxonomy ID	29065	
BioProject	PRJEB65269	
BioSample ID	Genome sequencing: SAMEA14448654 Hi-C scaffolding: SAMEA14448646 RNA sequencing: SAMEA14448654	
Isolate information	mMarMar1, male: liver (genome sequence), muscle (Hi-C sequencing), liver (RNA sequencing)	
Assembly metrics *		*Benchmark*
Consensus quality (QV)	63.6	*≥ 50*
*k*-mer completeness	100.0%	*≥ 95%*
BUSCO **	C:95.2%[S:94.3%,D:0.9%], F:0.9%,M:3.9%,n:14,502	*C ≥ 95%*
Percentage of assembly mapped to chromosomes	98.23%	*≥ 95%*
Sex chromosomes	XY	*localised* *homologous pairs*
Organelles	Mitochondrial genome: 16.57 kb	*complete single* *alleles*
Raw data accessions		
PacificBiosciences Sequel IIe	ERR11867241, ERR11867238, ERR11867240, ERR11867239	
Hi-C Illumina	ERR11872608	
PolyA RNA-Seq Illumina	ERR11872609	
Genome assembly		
Assembly accession	GCA_963455335.1	
*Accession of alternate* *haplotype*	GCA_963455355.1	
Span (Mb)	2,484.6	
Number of contigs	2727	
Contig N50 length (Mb)	1.6	
Number of scaffolds	476	
Scaffold N50 length (Mb)	146.3	
Longest scaffold (Mb)	217.42	

* Assembly metric benchmarks are adapted from column VGP-2020 of “Table 1: Proposed standards and metrics for defining genome assembly quality” from Rhie *et al.* (2021). ** BUSCO scores based on the carnivora_odb10 BUSCO set using version 5.4.3. C = complete [S = single copy, D = duplicated], F = fragmented, M = missing, n = number of orthologues in comparison. A full set of BUSCO scores is available at https://blobtoolkit.genomehubs.org/view/Martes%20martes/dataset/CAUOHJ01/busco.

**Table 2.** Chromosomal pseudomolecules in the genome assembly of *Martes martes*, mMarMar1

INSDC accession	Chromosome	Length (Mb)	GC%
OY734063.1	1	217.42	41.0
OY734064.1	2	206.14	39.5
OY734065.1	3	196.26	39.5
OY734066.1	4	191.18	42.0
OY734067.1	5	165.75	41.0
OY734068.1	6	148.92	40.5
OY734069.1	7	146.29	42.5
OY734070.1	8	142.36	41.5
OY734071.1	9	139.28	43.5
OY734073.1	10	116.35	43.5
OY734074.1	11	108.09	42.0
OY734075.1	12	96.3	43.5
OY734076.1	13	93.66	43.5
OY734077.1	14	90.3	44.0
OY734078.1	15	69.26	43.0
OY734079.1	16	65.21	47.5
OY734080.1	17	59.81	47.0
OY734081.1	18	41.22	50.5
OY734072.1	X	122.26	40.5
OY734082.1	Y	19.85	52.5
OY734083.1	MT	0.02	42.5

The estimated Quality Value (QV) of the final assembly is 63.6 with *k*-mer completeness of 100.0%, and the assembly has a BUSCO v completeness of 95.2% (single = 94.3%, duplicated = 0.9%), using the carnivora_odb10 reference set ( *n* = 14,502).

Metadata for specimens, barcode results, spectra estimates, sequencing runs, contaminants and pre-curation assembly statistics are given at https://links.tol.sanger.ac.uk/species/29065.

## Methods

### Sample acquisition and nucleic acid extraction

An adult male *Martes martes* (specimen ID NHMUK014446401, ToLID mMarMar1) was collected on 2021-09-09, when it was found freshly killed as a road traffic accident victim on the A890 next to coniferous woodland in Glen Carron (57.51, –5.21). The specimen was collected and identified by David O'Brien (NatureScot) and preserved by dry freezing at –80 °C.

The workflow for high molecular weight (HMW) DNA extraction at the Wellcome Sanger Institute (WSI) Tree of Life Core Laboratory includes a sequence of core procedures: sample preparation; sample homogenisation, DNA extraction, fragmentation, and clean-up. In sample preparation, the mMarMar1 sample was weighed and dissected on dry ice ( Jay *et al.*, 2023). For sample homogenisation, liver tissue was cryogenically disrupted using the Covaris cryoPREP ^®^ Automated Dry Pulverizer ( Narváez-Gómez *et al.*, 2023). HMW DNA was extracted using the Manual MagAttract v1 protocol ( Strickland *et al.*, 2023b). DNA was sheared into an average fragment size of 12–20 kb in a Megaruptor 3 system with speed setting 30 ( Todorovic *et al.*, 2023). Sheared DNA was purified by solid-phase reversible immobilisation ( Strickland *et al.*, 2023a): in brief, the method employs a 1.8X ratio of AMPure PB beads to sample to eliminate shorter fragments and concentrate the DNA. The concentration of the sheared and purified DNA was assessed using a Nanodrop spectrophotometer and Qubit Fluorometer and Qubit dsDNA High Sensitivity Assay kit. Fragment size distribution was evaluated by running the sample on the FemtoPulse system.

RNA was extracted from liver tissue of mMarMar1 in the Tree of Life Laboratory at the WSI using the RNA Extraction: Automated MagMax™ *mir*Vana protocol ( do Amaral *et al.*, 2023). The RNA concentration was assessed using a Nanodrop spectrophotometer and a Qubit Fluorometer using the Qubit RNA Broad-Range Assay kit. Analysis of the integrity of the RNA was done using the Agilent RNA 6000 Pico Kit and Eukaryotic Total RNA assay.

Protocols developed by the WSI Tree of Life laboratory are publicly available on protocols.io ( Denton *et al*., 2023).

### Sequencing

Pacific Biosciences HiFi circular consensus DNA sequencing libraries were constructed according to the manufacturers’ instructions. Poly(A) RNA-Seq libraries were constructed using the NEB Ultra II RNA Library Prep kit. DNA and RNA sequencing was performed by the Scientific Operations core at the WSI on Pacific Biosciences Sequel IIe (HiFi) and Illumina NovaSeq 6000 (RNA-Seq) instruments. Hi-C data were also generated from muscle tissue of mMarMar1 using the Arima v2 kit. The Hi-C sequencing was performed using paired-end sequencing with a read length of 150 bp on the Illumina NovaSeq 6000 instrument.

### Genome assembly and curation

Assembly was carried out with Hifiasm ( Cheng *et al.*, 2021) and haplotypic duplication was identified and removed with purge_dups ( Guan *et al.*, 2020). The assembly was then scaffolded with Hi-C data ( Rao *et al.*, 2014) using YaHS ( Zhou *et al.*, 2023). The assembly was checked for contamination and corrected using the TreeVal pipeline ( Pointon *et al.*, 2023). Manual curation was performed using JBrowse2 ( Diesh *et al.*, 2023), HiGlass ( Kerpedjiev *et al.*, 2018) and PretextView ( Harry, 2022). The mitochondrial genome was assembled using MitoHiFi ( Uliano-Silva *et al.*, 2023), which runs MitoFinder ( Allio *et al.*, 2020) or MITOS ( Bernt *et al.*, 2013) and uses these annotations to select the final mitochondrial contig and to ensure the general quality of the sequence.

### Evaluation of final assembly

A Hi-C map for the final assembly was produced using bwa-mem2 ( Vasimuddin *et al.*, 2019) in the Cooler file format ( Abdennur & Mirny, 2020). To assess the assembly metrics, the *k*-mer completeness and QV consensus quality values were calculated in Merqury ( Rhie *et al.*, 2020). This work was done using Nextflow ( Di Tommaso *et al.*, 2017) DSL2 pipelines “sanger-tol/readmapping” ( Surana *et al.*, 2023a) and “sanger-tol/genomenote” ( Surana *et al.*, 2023b). The genome was analysed within the BlobToolKit environment ( Challis *et al.*, 2020) and BUSCO scores ( Manni *et al.*, 2021; Simão *et al.*, 2015) were calculated.

Table 3 contains a list of relevant software tool versions and sources.

**Table 3.** Software tools: versions and sources.

Software tool	Version	Source
BlobToolKit	4.2.1	https://github.com/blobtoolkit/blobtoolkit
BUSCO	5.3.2	https://gitlab.com/ezlab/busco
Hifiasm	0.16.1-r375	https://github.com/chhylp123/hifiasm
HiGlass	1.11.6	https://github.com/higlass/higlass
Merqury	MerquryFK	https://github.com/thegenemyers/MERQURY.FK
MitoHiFi	2	https://github.com/marcelauliano/MitoHiFi
PretextView	0.2	https://github.com/wtsi-hpag/PretextView
purge_dups	1.2.3	https://github.com/dfguan/purge_dups
sanger-tol/genomenote	v1.0	https://github.com/sanger-tol/genomenote
sanger-tol/readmapping	1.1.0	https://github.com/sanger-tol/readmapping/tree/1.1.0
YaHS	yahs-1.1.91eebc2	https://github.com/c-zhou/yahs

### Wellcome Sanger Institute – Legal and Governance

The materials that have contributed to this genome note have been supplied by a Darwin Tree of Life Partner. The submission of materials by a Darwin Tree of Life Partner is subject to the **‘Darwin Tree of Life Project Sampling Code of Practice’**, which can be found in full on the Darwin Tree of Life website here. By agreeing with and signing up to the Sampling Code of Practice, the Darwin Tree of Life Partner agrees they will meet the legal and ethical requirements and standards set out within this document in respect of all samples acquired for, and supplied to, the Darwin Tree of Life Project.

Further, the Wellcome Sanger Institute employs a process whereby due diligence is carried out proportionate to the nature of the materials themselves, and the circumstances under which they have been/are to be collected and provided for use. The purpose of this is to address and mitigate any potential legal and/or ethical implications of receipt and use of the materials as part of the research project, and to ensure that in doing so we align with best practice wherever possible. The overarching areas of consideration are:

•      Ethical review of provenance and sourcing of the material

•      Legality of collection, transfer and use (national and international)

Each transfer of samples is further undertaken according to a Research Collaboration Agreement or Material Transfer Agreement entered into by the Darwin Tree of Life Partner, Genome Research Limited (operating as the Wellcome Sanger Institute), and in some circumstances other Darwin Tree of Life collaborators.

## Data Availability

European Nucleotide Archive: *Martes martes* (European pine marten). Accession number PRJEB65269; https://identifiers.org/ena.embl/PRJEB65269 ( Wellcome Sanger Institute, 2023). The genome sequence is released openly for reuse. The *Martes martes* genome sequencing initiative is part of the Darwin Tree of Life (DToL) project. All raw sequence data and the assembly have been deposited in INSDC databases. The genome will be annotated using available RNA-Seq data and presented through the Ensembl pipeline at the European Bioinformatics Institute. Raw data and assembly accession identifiers are reported in Table 1.

## Author information

Members of the Natural History Museum Genome Acquisition Lab are listed here: https://doi.org/10.5281/zenodo.7139035.

Members of the Darwin Tree of Life Barcoding collective are listed here: https://doi.org/10.5281/zenodo.4893703.

Members of the Wellcome Sanger Institute Tree of Life Management, Samples and Laboratory team are listed here: https://doi.org/10.5281/zenodo.10066175.

Members of Wellcome Sanger Institute Scientific Operations: Sequencing Operations are listed here: https://doi.org/10.5281/zenodo.10043364.

Members of the Wellcome Sanger Institute Tree of Life Core Informatics team are listed here: https://doi.org/10.5281/zenodo.10066637.

Members of the Tree of Life Core Informatics collective are listed here: https://doi.org/10.5281/zenodo.5013541.

Members of the Darwin Tree of Life Consortium are listed here: https://doi.org/10.5281/zenodo.4783558.
