# Supplementary material for: The genome sequence of the European pine marten, Martes martes (Linnaeus, 1758)
Source: Wellcome Open Res. 2024 Jun 21;9:325. [Version 1] doi: 10.12688/wellcomeopenres.22458.1 (PMC11372345; doi:10.12688/wellcomeopenres.22458.1)
Supplement: Peer review history

# Reviewer report: Reviewer response for version 1

**DOI:** 10.21956/wellcomeopenres.24745.r93015

**Published:** 2024-09-04

**Authors:** Zsófia Fekete

**Affiliations:** 1 Department of Environmental and Biological Sciences, University of Eastern Finland, Joensuu, Finland

The article "The genome sequence of the European pine marten, *Martes martes* (Linnaeus, 1758)" describes the genome assembly of the European pine marten. The species is critically endangered in parts of Britain, where its current population is low despite reintroduction efforts and stable and viable populations present in certain areas. The authors have created a high quality, chromosome level assembly of the species which can help in supporting conservation efforts.

 The authors used appropriate techniques corresponding to current best practices. Both the sequencing and assembly approaches are adequate, although the options used with each software are not specified. Mentioning these would help reproducibility.

 2.48 Gbp long genome sequence is ordered into 20 chromosomal sequences, including the X and Y chromosomes, which corresponds to the known karyotype of *Martes martes*. The full assembly includes the mitochondrial DNA sequence as well. The plots and tables show the quality of the genome assembly well, and the sequence itself is readily available online with links provided in the article.

**Suggestions:**

- Mention specific parameters for the assembly where it is different from the default of the software used, or otherwise that default values and settings were used.

In summary, the article presents a high quality genome assembly of the European pine marten, a species that did not have a readily available reference genome before. The work is up to current standards.

Are sufficient details of methods and materials provided to allow replication by others?

Partly

Is the rationale for creating the dataset(s) clearly described?

Yes

Are the datasets clearly presented in a useable and accessible format?

Yes

Are the protocols appropriate and is the work technically sound?

Yes

Reviewer Expertise:

Bioinformatics, Genomics: Genome assemblies, population genomics, bioinformatics methods using various long- and short read sequencing types.

I confirm that I have read this submission and believe that I have an appropriate level of expertise to confirm that it is of an acceptable scientific standard.

# Reviewer report: Reviewer response for version 1

**DOI:** 10.21956/wellcomeopenres.24745.r93008

**Published:** 2024-09-03

**Authors:** Giulio Formenti

**Affiliations:** 1 Vertebrate Genome Laboratory, The Rockefeller University, New York, NY, USA

- The marten is iconic also for other European countries (eg. Italy), I suggest rephrasing. If there is something of this particular individual/population specific to UK, it should be stated.
- Citation The Wildlife Trusts, no date probably requires editing.
- It would be interesting to know a little bit how the new assembly compares to the previous ones for same/closely-related species.

Long live the pine marten!

Are sufficient details of methods and materials provided to allow replication by others?

Yes

Is the rationale for creating the dataset(s) clearly described?

Yes

Are the datasets clearly presented in a useable and accessible format?

Yes

Are the protocols appropriate and is the work technically sound?

Yes

Reviewer Expertise:

Genomics, biology

I confirm that I have read this submission and believe that I have an appropriate level of expertise to confirm that it is of an acceptable scientific standard.

# Reviewer report: Reviewer response for version 1

**DOI:** 10.21956/wellcomeopenres.24745.r93010

**Published:** 2024-08-12

**Authors:** Graham Etherington

**Affiliations:** 1 Earlham Institute, Norwich, UK

The manuscript introduces the history and status of the Pine marten in the UK and sets out a rationale for the need of a genome sequence for this species, which can inform on conservation genomics work.

**The methods and results are well set out and explained and I only have a couple of minor comments:**

 1. The sentence ending "with 19% of woodland found in Scotland" is a bit confusing. Are you separating Scotland from the UK (so 19% of Scotland is woodland), or is 19% of UK woodland found in Scotland?

 2. The following sentence doesn't make sense - please reword.

Are sufficient details of methods and materials provided to allow replication by others?

Yes

Is the rationale for creating the dataset(s) clearly described?

Yes

Are the datasets clearly presented in a useable and accessible format?

Yes

Are the protocols appropriate and is the work technically sound?

Yes

Reviewer Expertise:

Genome assembly, conservation genomics, avian and mustelid genomics.

I confirm that I have read this submission and believe that I have an appropriate level of expertise to confirm that it is of an acceptable scientific standard.
